# Recycling of worn out brake pads ‒ impact on tribology and environment

**DOI:** 10.1038/s41598-020-65265-w

**Published:** 2020-05-20

**Authors:** Yezhe Lyu, Jijie Ma, Anna Hedlund Åström, Jens Wahlström, Ulf Olofsson

**Affiliations:** 1 Department of Machine Design, KTH Royal Institute of Technology, SE-10044 Stockholm, Sweden; 2 College of Engineering, Zhejiang Normal University, 321004 Jinhua, China; 3 Department of Mechanical Engineering, Lund University, SE-22100 Lund, Sweden

**Keywords:** Environmental impact, Sustainability, Mechanical engineering, Composites

## Abstract

Disc brake systems are widely used on commercial vehicles for braking. The brake pads are usually replaced by new ones before being totally worn out. Current methods to deal with the replaced brake pads include landfill and combustion, resulting in a huge waste of resources and increase of CO_2_ footprint. From a sustainable point of view, this study aims to evaluate the feasibility of recycling replaced brake pads by addressing a protocol recycling procedure. The results show that the recycled brake pads yield similar friction, wear and airborne particle emission to virgin brake pads. A streamlined life cycle assessment is conducted to compare the environmental impacts between producing virgin brake pads and recycling replaced brake pads. Energy consumption and CO_2_ footprint of the recycled brake pads are 36% and 34% less than virgin brake pads, indicating that recycling could be a promising method of handling replaced brake pads.

## Introduction

**Figure 1:**
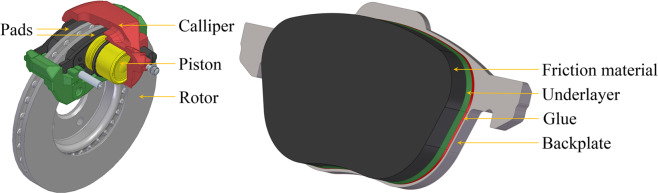
Typical components of a disc brake system and different layers of the brake pad.

Disc brake systems are widely used on cars and commercial vehicles for braking. A disc brake system is basically composed of a calliper with one or more pistons, a brake rotor, and two brake pads. The brake rotor is usually made from grey cast iron, which has a high thermal conductivity. The brake pads typically consist of a stiff back plate on which a friction material is bonded (Fig. 1). The friction material can be classified as non-asbestos organic, non-metallic, low-metallic, semi-metallic, or ceramic. In Europe, most passenger cars use low-metallic brake pads, which are complex in constitution and contain several different ingredients as reinforcing fibres, binders, fillers, and frictional additives^1^. During braking, rotor slides against the friction materials, transforming the kinetic energy of the vehicle into frictional heat^2,3^. In such a way, the brake pads and brake rotors slowly wear out. During braking, some of the worn materials deposit on roadways and the others become airborne as particulate matter. It has been found that a large proportion (20% to 50%) of the worn materials during braking become airborne particles in road transport^4,5^. The micro- and nano-size airborne particles can easily transport for several kilometres in the ambient environment^6^, inducing short-term^7^ and long-term^8^ adverse effects on lung function and weakening pulmonary antimicrobial immune defence^9^.

Brake pads and rotors are usually replaced by new ones before they are totally worn out. One study^10^ estimated that there is a need of 16 spare pairs of pads and four rotors during the entire lifetime of a car. Since 2010, more than 70 million cars and commercial vehicles are produced every year and it is estimated that one billion cars are running on roads all over the world^11^. Under such circumstances, replaced brake pads are accumulating rapidly. Since the friction materials on brake pads are non-degradable, disposal of the replaced brake pads is difficult. A common way for dealing with the replaced brake pads is landfill. This method is prone to induce heavy metal pollution of soils^12^. Another popular way is to combust them to extract the internal energy. Neither of these two methods is advisable due to the waste of resources and increase of CO_2_ footprint. From a sustainable point of view, recycling replaced brake pads is a promising method due to the conservation of resources and the environment.

Replaced brake pad recycling is a new concept and relevant knowledge is still scarce. This paper aims to evaluate the feasibility of recycling replaced brake pads. This feasibility includes two aspects. One aspect concerns environmental impacts, i.e. the CO_2_ footprint and energy consumption during the recycling procedure. The other aspect is to evaluate if the coefficient of friction (CoF), wear and airborne particle emission of the recycled brake pads is comparative to the newly manufactured virgin brake pads. Accordingly, this paper firstly tested a commercial brake pad friction material with regard to its CoF, wear and airborne particle emission in a pin-on-disc tribometer. Afterwards, the tested friction material is put into a specific recycling procedure. Finally, the CoF, wear and airborne particle emission of recycled friction materials are evaluated with the same test condition as the virgin friction material. A streamlined life cycle assessment (LCA) of the recycling procedure is conducted to identify the environmental impacts.

## Results

### Test plan

The tribological experiment used a pin-on-disc tribometer to test pin samples extracted from virgin and recycled brake pad friction materials against disc samples cut from commercial grey cast iron brake rotors. Two sliding speeds and two contact pressures at the pin-disc contact are tested in the experiment. The sliding speeds and contact pressures correspond to typical values of urban traffic conditions^1,13^. This test plan forms a full 2^3^ factorial design, yielding eight facts (2 levels × 3 factors) as shown in Table 1. Each fact has three replicate runs. The response variables of the experiment include CoF, wear loss, and particle generation rate.

**Table 1:** Full 2^3^ factorial design for the response variables.

Factors	Sliding speed (S)	Pressure (P)	Material (M)
+	2 m/s	1.2 MPa	Virgin
−	1 m/s	0.3 MPa	Recycled

### Friction

**Figure 2:**
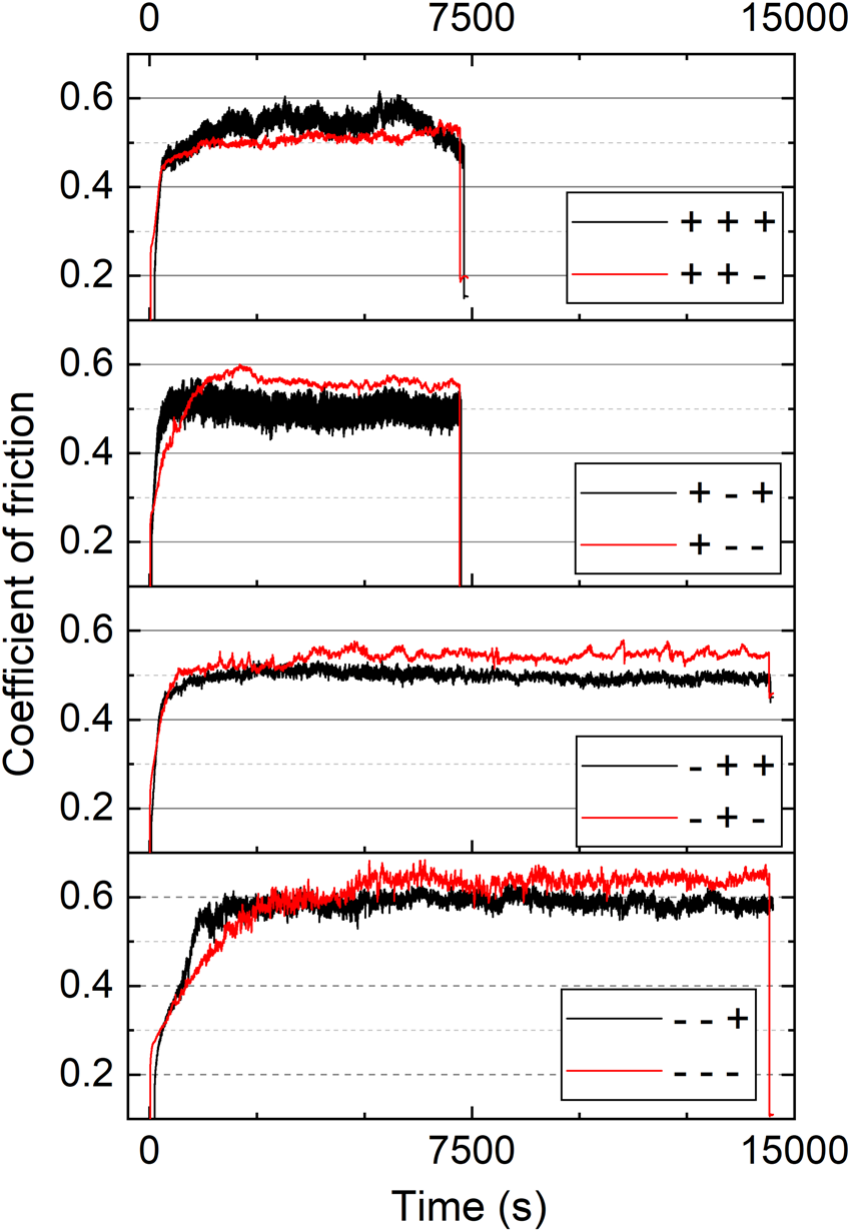
Time history of the measured CoF at eight factorial designed facts according to Table 1.

The ambient temperature and relative humidity in the tribology test were not controlled but measured before and after each test. The ambient temperature varied between 24 °C and 27 °C, and relative humidity between 23% and 42%. Figure 2 shows the representative curves of the measured CoF at varied sliding speed and contact pressure levels. The factor levels in Fig. 2 keep consistency to the design in Table 1, i.e. from left to right are S, P, M. It can be noted that all tests yield a running-in period before reaching the steady state, in which the friction keeps stable to the end of the test.

### Particle concentration

**Figure 3:**
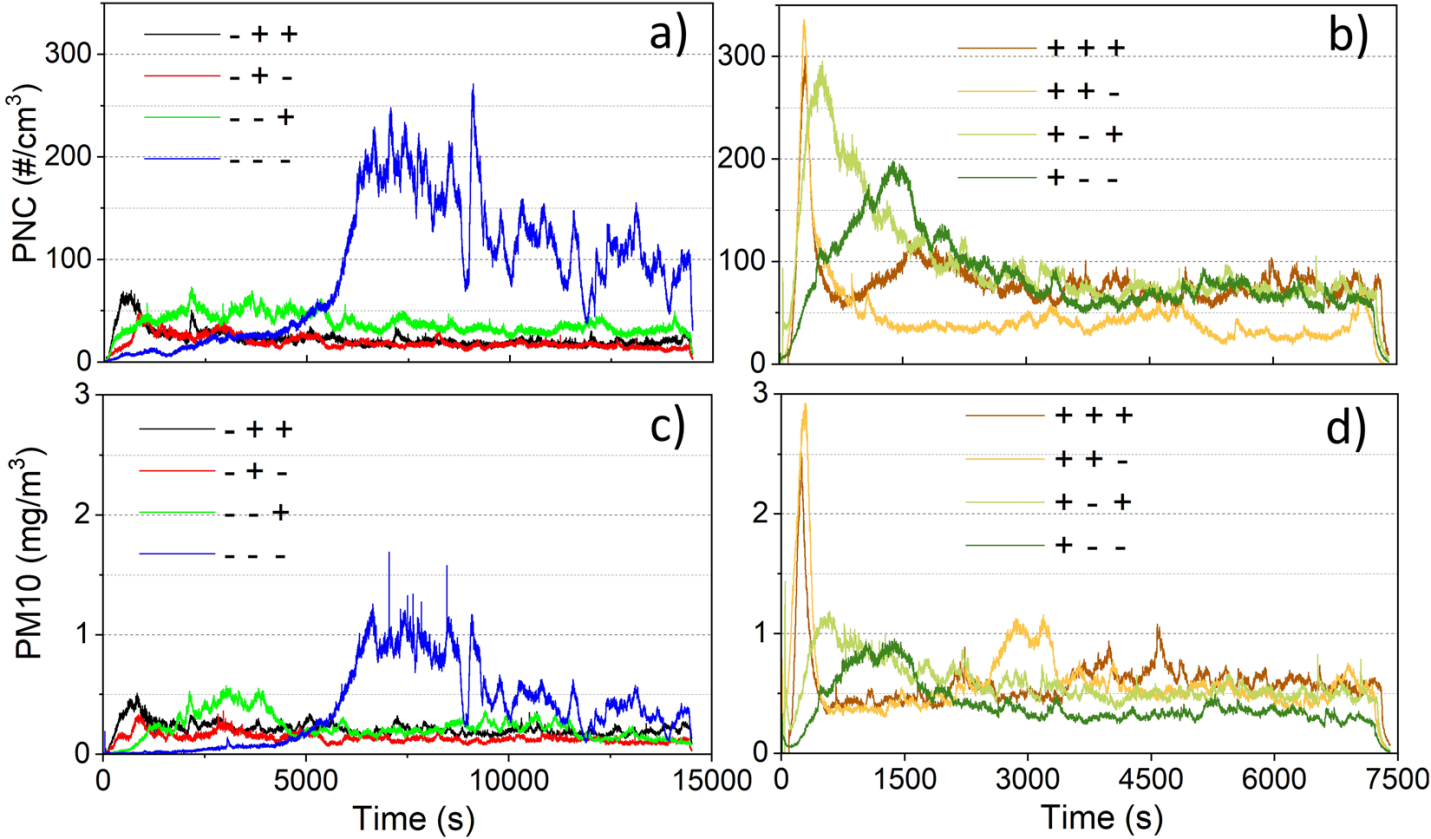
Time history of the measured PNC (**a,b**) and PM10 (**c,d**) at eight factorial designed facts according to Table 1.

Representative curves of the measured particle number concentration (PNC) by TSI Optical Particle Sizer model 3330 (OPS) and PM10 (particulate matter with an aerodynamic diameter smaller than 10 μm) measured by DEKATI Electrical Low-Pressure Impactor (ELPI+) versus time are presented in Fig. 3. It can be noted that most of the tests show a running-in period before reaching the steady state similar to the friction curves in Fig. 2. The running-in period is the process that the contacting surfaces get engaged with each other by removing the initial surface texture on the superficial surfaces. After the running-in, the steady state is reached where the contacting surfaces have conformal contact, so that the friction and airborne particle emission curves become stable. One exception is seen on the test on a recycled sample at 1 m/s and 0.3 MPa (blue curves in Fig. 3a,c), where the running-in period seems to postpone and last to the end of the test.

### Environmental impacts

The environmental impacts of both the virgin and recycled brake pads are assessed in terms of CO_2_ footprint and energy consumption for the entire life cycle consisting of several stages, i.e. material, manufacture, transport, use and disposal. The manufacture stage is streamlined, in which some common machining procedures for the virgin and recycled pads are identical. Landfill is selected as the method for disposal of the replaced virgin and recycled brake pads. The calculated data is shown in Table 2.

**Table 2:** Environmental impacts, i.e. CO_2_ footprint and energy consumption of virgin and recycled brake pads for the entire life cycle.

Stage		Material	Manufacture (streamlined)	Transport	Use	Disposal	Total
CO_2_ (g)	Recycled	149	0.23	2.93	691	2.63	845.79
	Virgin	598	0.23	2.93	691	2.63	1294.79
Energy (MJ)	Recycled	2.62	0.003	0.04	9.60	0.04	12.30
	Virgin	9.70	0.003	0.04	9.60	0.04	19.38

## Discussion

In order to evaluate the feasibility of recycling worn-out brake pads, the first criterion to be fulfilled is that the recycled brake pad materials should possess similar brake performance (i.e. CoF, wear and particle generation rate) as the virgin brake pad materials. The 2^3^ factorial design for the tribological experiment has three factors, and each factor has two levels. In order to evaluate how the change in the factor influences the response variables, the “effect” of the factor is calculated. The “effect” of a factor is the change in the response variables as the factor moves from the “−” level to the “+” level. Sometimes, the factors do not behave independently and are therefore said to “interact”^14^. Due to that three replicate runs were conducted for each test condition; the estimated effects and standard errors can be obtained as shown in Table 3. By comparing the estimated effects with their standard errors, some items that require interpretation are highlighted in italics with brackets. Other effects, however, remain unknown since they could be generated by the noise. It should be noted that the interaction effects should be equally interpreted as the main effects.

**Table 3:** Calculated effects and standard errors for the 2^3^ factorial design.

Effect	Factors	CoF	Pin wear (mg)	Disc wear (mg)	Particle number rate (10^4^#/m)	Particle mass rate (μg/m)
		Estimate ± standard error				
Average		0.55 ± 0.01	90 ± 9	53 ± 6	14 ± 4	0.64 ± 0.13
Main effects	Material (M)	−0.02 ± 0.02	*(42* ± *18)*	16 ± 12	−12 ± 8	−0.34 ± 0.26
	Speed (S)	*(−0.05* ± *0.02)*	−21 ± 18	*(40* ± *12)*	−12 ± 8	−0.12 ± 0.26
	Pressure (P)	*(−0.06* ± *0.02)*	*(67* ± *18)*	−13 ± 12	−17 ± 8	−0.23 ± 0.26
Two-factor interactions	M × S	0.01 ± 0.02	−11 ± 18	5 ± 12	15 ± 8	*(0.64* ± *0.26)*
	M × P	0.001 ± 0.02	25 ± 18	18 ± 12	14 ± 8	*(0.62* ± *0.26)*
	S × P	*(0.04* ± *0.02)*	−5 ± 18	*(31* ± *12)*	15 ± 8	*(0.72* ± *0.26)*
Three-factor interaction	M × S × P	0.001 ± 0.02	−11 ± 18	2 ± 12	−14 ± 8	−0.31 ± 0.26

The factors speed (S) and pressure (P) have the greatest effects on the CoF, and their interaction effect has a similar influence. The factor material (M) seems not to strongly affect the CoF. However, its effect on the pin wear is notable and runs second, just after the factor pressure (P). The factor speed (S) dominates the change in disc wear and shows a strong interaction with factor pressure (P). For the particle number rate, all the main effects and interaction effects are likely to contribute equally and it is difficult to distinguish the dominant ones. The three two-factor interactions have stronger effects on particle mass rate than the individual main effects.

The result of most practical interest is that the factor material (M) is only found to affect the pin wear, and its effects on other response variables are not noticeable. It should also be noted that the main effect of factor material (M) on pin wear is positive, indicating that the recycled pin sample (− level in the design) has lower wear loss than the virgin pin sample (+ level in the design). In other words, the recycling procedure did not degrade the performance of the brake pad material regarding CoF, wear and particle generation rate.

The other required criterion is that the environmental impacts (i.e. energy consumption and CO_2_ footprint) of recycling the worn-out brake pads should not be higher than producing a new one. From Table 2, the energy consumption and CO_2_ footprint of the recycled brake pads are 36% and 34% less than the virgin brake pads, respectively. It should be noted that the only difference between the recycled and virgin brake pads falls in the material stage. In other stages, the recycled and virgin brake pads yield identical environmental impacts. Accordingly, an elaborate investigation into the material stage is conducted to analyse the causes of the reduced energy consumption and CO_2_ footprint.

**Figure 4:**
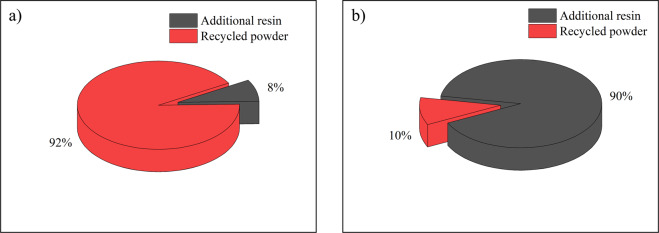
Mass percentage (**a**) and energy consumption share (**b**) of the recycled brake pad material.

The embodied energy of the recycled powder in this study comes from the recycling procedures step 1 and 2 (Table 5). Other steps, as common procedures for both recycled and virgin pins, are not included. Figure 4 demonstrates the mass and energy share of the additional resin and recycled powder in the recycled brake pad materials. Recycled material accounts for 92% of the total weight but consumes only 10% of the energy in the material phase. It is expected to have a lower energy share by taking this protocol procedure into mass production.

To get a better view of the potential decrease in energy consumption and CO_2_ footprint from the systems perspective, the figures can be connected to the EU car fleet. The numbers of passenger cars changes per year, but an average between 2012 and 2016 is calculated at 246 million cars per year^15^. Usually a car has four disc brakes, i.e. eight brake pads in total. Assuming the following scenario for recycling:

- Life length of car 240,000 km
- All pads changed four times
- At least 25% of the pads are recycled

This results in 1,968 million pads for material recycling, saving 13.9 billion MJ of energy or 6.2 million kg CO_2_ when comparing virgin produced pads with recycled pads, see Table 2. The energy figure can be related to energy consumption for heating and hot water in around 300,000 one- and two dwelling buildings in the year 2017 in Sweden^16^. The emission of CO_2_ can be compared to the average emissions, 118.5 g/km from new passenger cars in the EU in 2017^17^, resulting in a transport length comparable to 1,300 times around the Earth. To be able to reuse the recycled brake pads into new pads, the material constitution of the friction layer needs to be known. Therefore, the traceability is of great importance and a specific infrastructure for treatment with collecting, sorting etc. must be developed. Another more direct solution is to use this recycled friction material as an underlayer material for production of new pads.

## Methods

### Experimental set-up

**Figure 5:**
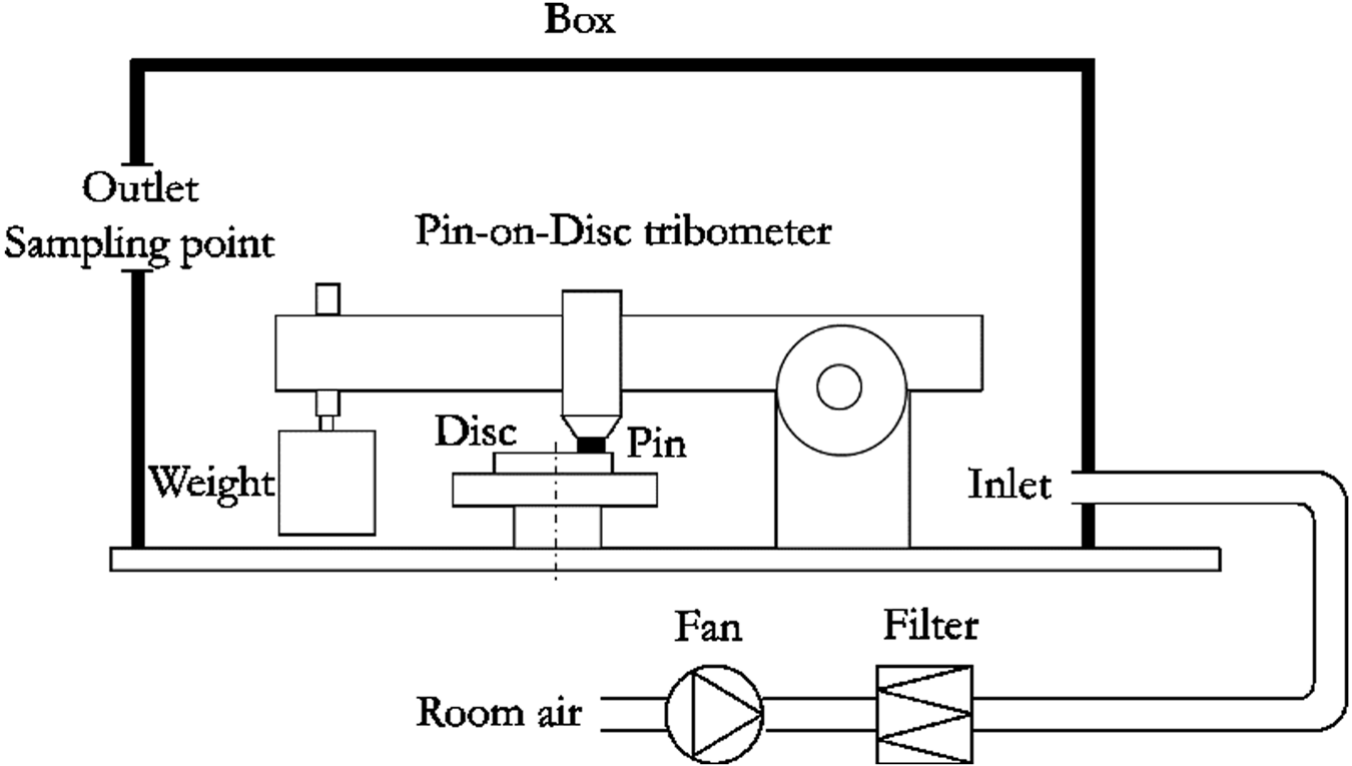
Schematic of the test equipment.

The experiment is performed in a pin-on-disc tribometer placed in a one-way ventilated chamber^18^. Figure 5 shows the schematic of the test set-up. The fan pumps in the ambient air from the room air opening and pushes it through a nano-size particle filter (particle collection efficiency of 99.95%). Plastic tubes are totally sealed. In such a way, theoretically clean air without background airborne particles can be achieved in the closed box chamber. The inlet air velocity is measured with a TSI air velocity transducer Model 8455 so that the air flow rate can be calculated with the known area of the air inlet. During testing, the vertically mounted pin sample slides with a horizontally rotating disc. The dead weight applies the normal force at the pin-disc contact. An HBM Z6FC3/20 kg load cell is set on the side of the pin holder and measures the tangential force at the contact. CoF between the pin-disc contact can be calculated by dividing the measured tangential force by the normal force. Airborne particles generated from the sliding pin-disc contact is measured by a TSI Optical Particle Sizer (OPS) model 3330 and a DEKATI Electrical Low-Pressure Impactor (ELPI+) set at the air outlet sampling point. The OPS measures particle number concentration and size distribution in the size range from 0.3 μm to 10 μm. The ELPI+ measures particle mass concentration and size distribution in the size range from 6 nm to 10 μm. The sampling frequency is 1 Hz for all instruments. The mass loss of samples is measured by weighing them before and after the test to the nearest 0.1 mg using a Sartorius ME614S balance.

### Materials

Commercial brake pad friction material and brake rotor material used on a typical medium sized car in the EU are tested in this study. This disc brake system consists of a sliding caliper, two low-metallic pads and a ventilated grey cast iron rotor. The chemical compositions (obtained with X-ray Fluorescence Spectrometer) of the friction material on the brake pad and grey cast iron rotor are shown in Table 4. Pin samples (10 mm in diameter for the testing surface) are cut from unused virgin brake pads and disc samples (60 mm in diameter and 6 mm thick) from unused grey cast iron brake rotors.

**Table 4:** Chemical compositions (wt.%) of the brake pad friction material and cast-iron brake rotor.

Element	C	Si	Mn	S	Fe	Cu	Zn	Sn	Ca	Cr	Mg	Al	F
Pad	39.8	3.13	N/A	2.37	16.7	9.12	5.57	4.08	0.52	2.53	6.71	7.31	0.43
Disc	3.80	1.80	0.65	0.06	balance	N/A	N/A	N/A	N/A	N/A	N/A	N/A	N/A

### Tribology experiment

Two sliding speeds (2 m/s and 1 m/s) between the pin and disc are tested. These two speeds correspond to typical city traffic conditions^1^, where the corresponding mean vehicle speeds are 20 km/h and 10 km/h, respectively. Each test yields a same sliding distance of 14.1 km. Two contact pressures (1.2 MPa and 0.3 MPa) are tested, which correspond to hard braking and decelerating to correct speed conditions. Each contact combination (sliding speed + contact pressure) is repeated three times.

### Recycling procedure

The tested virgin pin samples are recycled following the process shown in Table 5. It should be noted that in the screening step, some crushed powders larger than 1800 μm are screened out so that 8 wt.% phenolic resin is added in step 3 to maintain the same amount of recycled pin samples.

**Table 5:** Recycling procedure of the tested virgin pins.

Step	Action	Equipment	Parameter	Duration (min)
1	Crushing	Ball milling machine	50 W	10
2	Screening	Manual sieve in 1800 μm	N/A	N/A
3	Adding 8 wt.% phenolic resin	Manual	N/A	N/A
4	Mixing	Turbula Shaker-Mixer	180 W	15
5	Hot pressing	Hot presser	150 °C/15 MPa	7
6	Curing	Industrial oven	200 °C	600

### Life cycle assessment

The environmental impact of brake pads mainly comes from the following stages: material stage, manufacture stage, transport, use stage, and disposal^19^. This study takes energy consumption and CO_2_ footprint as the main indices to assess the environmental impact. The energy consumption of the five stages in lifecycle is calculated as below. The CO_2_ footprint is not addressed in detail due to its high correspondence to the energy consumption. The equations and parameters for both energy consumption and CO_2_ footprint are provided as a supplementary.

Material phase energy consumption refers to the embodied energy of the raw materials, which is related to the recycling percentage due to the fact that several raw materials involve recycling contents. Embodied energy of raw materials is the sum of virgin ingredients and the recycled ones. Due to the mass loss in manufacturing, the weight of raw materials is greater than the total output of the recycled brake pads. Thus, it is necessary to correct the mass loss according to the removed percentage. Depending how the manufacturing waste is treated, through energy recovery or material recycling, the credit will be different. The energy consumption in manufacture stage includes the processing of each raw material and the following machining of the semi-finished product as a whole. The mass correcting factor in the material phase is calculated according to the processing technique here and the cut off percentage. For raw material processes, the manufacture energy is the sum of all processes for each ingredient. Similarly, the manufacture energy for semi-finished product is the sum of the energy consumption in each process.

Both raw materials and the final product involve transport. In this study, transport energy consumption of raw materials is included in the material phase, leaving only the transport of final product to be calculated. Transport energy is in proportion with product mass and travel distance. The energy consumption of brake pads in the use stage depends on the vehicle type they are used on, and the life cycle travel distance together with the vehicle. Disposal mainly relates to the preparation work for landfill, which simply requires the collection of waste pads.

## Supplementary information

Supplementary information.
