# Supplementary material for: Recycling of worn out brake pads ‒ impact on tribology and environment
Source: Sci Rep. 2020 May 20;10:8369. doi: 10.1038/s41598-020-65265-w (PMC7239870; doi:10.1038/s41598-020-65265-w)
Supplement: Supplementary file 1 — Supplementary information. [file 41598_2020_65265_MOESM1_ESM.pdf]

LCA equations and parameters

Parameters and value:

E_{rm} — the energy consumption of raw materials;
E_w — the energy consumption of waste materials in manufacture;
E_m — the energy consumption in the material phase;
m — the mass of raw materials;
M_{cf} — the mass correcting factor to compensate the mass loss in manufacture;
H_v — the embodied energy constant of virgin raw materials;
H_{rc} — the embodied energy constant of the recycled part in raw materials;
R_c — the fraction of the recycled part in raw materials;
H_c — the energy constant of waste collection;
H_{rw} — the embodied energy constant of waste materials;
N — the total number of ingredients in the formulation;
Z_i — the number of processes for ingredient i;
H_R — the energy constant of a raw material manufacture process;
m_{Ri} — the mass of ingredient i;
$M_{cfRi,j}$ — the mass correction factor of process number j for ingredient i;
Y — the total process number of a semi-finished product;
p — the percentage of the cut-off materials in a process;
H_P — the energy constant of a semi-finished product manufacture process;
$Cf_j(m)$ — the mass conversion factor of the process number j of the semi-finished product;
E_{mfR} — the manufacture energy of raw materials;
E_{mfP} — the manufacture energy of the semi-finished product;
E_{mf} — the energy consumption in manufacture stage;
E_t — the energy consumption in transport stage;
n — the number of transports;
H_{tj} — the energy constant of transport number j;
D_j — the distance of transport number j;
E_u — the energy consumption in use stage;
H_u — the energy constant of use depending on the vehicles it is applied;
C_e — the energy equivalence for electric transporting tools depending on countries;
DL — the lifelong working days of the product;
D_{day} — the average moving distance per day of the transporting tool in life cycle;
E_d — the energy consumption of disposal;
EOL — the way of end-of-life;
E — the total energy consumption in lifecycle;
CF — the total CO_2 footprint in lifecycle;
α — the conversion factor between energy and CO_2;

CF_m, CF_{mf}, CF_{mf}, CF_u and CF_d are corresponding parameter of CO_2 footprint in the material, manufacture, transport, use and disposal phase, respectively. The detail parameters labeled ‘CF’ for CO_2 footprint has the same meaning as labeled with ‘E’ for energy consumption. For example, H_v means the energy constant of virgin raw materials, while CF_v represents the CO_2 constant of virgin raw materials.

Table 1: Key parameter values for LCA calculation.

<table>
<tr><td rowspan="4">Energy</td><td>parameters</td><td colspan="2">H_v (MJ/kg)</td><td colspan="2">H_{R1} (MJ/kg)</td><td>H_{R2} (MJ/kg)</td><td>H_c (MJ/kg)</td><td colspan="2">H_{rc} (MJ/kg)</td><td>H_{t1} (MJ/kg)</td><td>H_{t2} (MJ/kg)</td><td>H_u (MJ/kg)</td></tr>
<tr><td>Recycled powder</td><td colspan="2">0.92</td><td colspan="2">0</td><td>0.3</td><td>0.2</td><td colspan="2">0.92</td><td>0.35</td><td>0.82</td><td>1.7</td></tr>
<tr><td>Phenolic resin</td><td colspan="2">92.6</td><td colspan="2">0</td><td>0.3</td><td>0.2</td><td colspan="2">92.5</td><td>0.35</td><td>0.82</td><td>1.7</td></tr>
<tr><td>formula materials</td><td colspan="11">Not listed</td></tr>
<tr><td rowspan="4">CO_2</td><td>parameters</td><td colspan="2">CF_v (g/kg)</td><td colspan="2">CF_{R1} (g/kg)</td><td>CF_{R2} (g/kg)</td><td>CF_c (g/kg)</td><td colspan="2">CF_{rc} (g/kg)</td><td>CF_{t1} (g/kg)</td><td>CF_{t2} (g/kg)</td><td>CF_u (g/kg)</td></tr>
<tr><td>Recycled powder</td><td colspan="2">81</td><td colspan="2">0</td><td>23</td><td>14</td><td colspan="2">81</td><td>25</td><td>59</td><td>72</td></tr>
<tr><td>Phenolic resin</td><td colspan="2">4310</td><td colspan="2">0</td><td>23</td><td>14</td><td colspan="2">4210</td><td>25</td><td>59</td><td>72</td></tr>
<tr><td>formula materials</td><td colspan="11">Not listed</td></tr>
<tr><td rowspan="4">Common</td><td>parameters</td><td>R_c</td><td>m (g)</td><td>p_1</td><td>p_2</td><td>D_1 (km)</td><td>D_2 (km)</td><td>C_e</td><td>α</td><td>DL</td><td>D_{day} (km)</td><td>EOL</td></tr>
<tr><td>Recycled powder</td><td>0</td><td>145.5</td><td>5%</td><td>0</td><td>500</td><td>50</td><td>1</td><td>0.07</td><td>3000</td><td>15</td><td>landfill</td></tr>
<tr><td>Phenolic resin</td><td>0</td><td>12.7</td><td>5%</td><td>0</td><td>500</td><td>50</td><td>1</td><td>0.07</td><td>3000</td><td>15</td><td>landfill</td></tr>
<tr><td>formula materials</td><td colspan="11">Not listed</td></tr>
</table>

Equations:

$$E_{rm} = m \cdot M_{cf} \cdot (H_v \cdot (1 - R_c) + H_{rc} \cdot R_c) \tag{1}$$
$$E_w = m \cdot (M_{cf} - 1) \cdot (H_c + H_{rw}) \tag{2}$$
$$E_m = E_{rm} + E_w \tag{3}$$
$$E_{mfR} = \sum_{i=1}^{N} \sum_{j=1}^{Z_i} H_{Ri,j} \cdot m_{Ri} \cdot M_{cfRi,j} \tag{4}$$
$$M_{cfRi,j} = \prod_{k=1}^{Z_i+Y-j+1} \frac{1}{1-p_k} \tag{5}$$
$$E_{mfP} = \sum_{j=1}^{Y} H_{Pj} \cdot Cf_j(m) \cdot \prod_{k=1}^{Y-j+1} \frac{1}{1-p_k} \tag{6}$$
$$E_{mf} = E_{mfR} + E_{mfP} \tag{7}$$
$$E_t = \sum_{j=1}^{n} H_{tj} \cdot m \cdot D_j \tag{8}$$
$$E_u = H_u \cdot m \cdot C_e \cdot DL \cdot D_{day} \tag{9}$$
$$E_d = H_c \cdot m \tag{10}$$
$$E = E_m + E_{mf} + E_t + E_u + E_d \tag{11}$$
$$CF_m = m \cdot M_{cf} \cdot (CF_v \cdot (1 - R_c) + CF_{rc} \cdot R_c) + m \cdot (M_{cf} - 1) \cdot (CF_c + CF_{rw}) \tag{12}$$
$$CF_{mf} = \sum_{i=1}^{N} \sum_{j=1}^{Z_i} CF_{Ri,j} \cdot m_{Ri} \cdot \prod_{k=1}^{Z_i+Y-j+1} \frac{1}{1-p_k} + \sum_{j=1}^{Y} H_{Pj} \cdot Cf_j(m) \cdot \prod_{k=1}^{Y-j+1} \frac{1}{1-p_k} \tag{13}$$
$$CF_t = \sum_{j=1}^{n} CF_{tj} \cdot m \cdot D_j \tag{14}$$
$$CF_u = CF_u \cdot m \cdot C_e \cdot DL \cdot D_{day} \tag{15}$$
$$CF_d = \alpha \cdot E_d \tag{16}$$
$$CF = CF_m + CF_{mf} + CF_t + CF_u + CF_d \tag{17}$$
